# Kaposi’s Clinic

**Published:** 1894-10

**Authors:** Frank J. Thornbury

**Affiliations:** 469 Delaware Avenue; Buffalo, N. Y.


### KAPOSI'S CLINIC—INTERESTING DERMATOLOGICAL CASES.[1]

By FRANK J. THORNBURY, M. D., Buffalo, N. Y.

I HAD intended giving a somewhat extensive account of the clinic of Professor Kaposi and the opportunities which it presents for the study of skin diseases, but am obliged to limit this paper to a more abridged description of certain interesting cases which I had occasion to observe during my course of study in Vienna.

Notwithstanding the great opportunities afforded by the clinics of London and Paris, it is generally conceded that Vienna presents a number of cases of certain diseases which are seldom, if at all, seen in the aforesaid and other localities.

Prurigo, the various forms of pemphigus, rhinoscleroma, phases of lupus, lichen, the varieties of favus, parasitic skin affections, molluscum, etc., and sometimes leprosy may be studied in Vienna with due satisfaction. The disease to which I would refer par excellence as being common in Austria and well exemplified in the clinics of Vienna is prurigo. The French for a long time claimed that this disease did not exist at all in their country, but when Professor Kaposi went to Paris he was able to discern a number of cases of what he designated prurigo, being treated by his French colleagues as other affections. Undoubtedly, this same explana-

1. Read before the Surgical Section of the Buffalo Academy of Medicine, September 4, 1894.

tion of the purported absence of prurigo in other countries would hold good.

Much of what has been said in the foregoing with reference to Vienna may be applied to the dermatological clinic of Prof. Pick, in Prague, which is inferior to few others in Europe, and presents opportunities quite as good as Vienna, if not better, in the presence of a far less number of students.

For him who will study laboratory, microscopic or experimental dermatology, the clinic of Unna, in Hamburg, is undoubtedly the proper place. One of the latter accomplishments of this celebrity is to have isolated, cultivated and demonstrated by inoculation, three separate and distinct varieties of favus, cultures of which I have in my possession and here exhibit. They were recently obtained from Unna in Hamburg.

One of the first cases to which I will refer as being of probable interest is a case of lichen rubor planus complicated by pemphigus vulgaris. A male patient, aged 50 years, suffering from the former disease, under treatment in one of the wards of Kaposi's clinic, suddenly became profoundly ill and presented a vesicular eruption, from which blebs soon formed. These contained a yellowish, albuminous and slightly alkaline fluid. The eruption showed an early tendency toward serpiginous arrangement, and was present not only over the lichen rubor areas, but also involved the previously unaffected portions of the skin, especially the anterior thoracic and abdominal regions, where large bullæ, containing purulent fluid, formed.

The patient was extremely prostrated, had a temperature for several days ranging from 39 to 41° C., and suffered from anorexia, insomnia and diarrhea. The lichen rubor with which he had been affected, and of which this was the second or third attack, was of the ordinary type. A mahogany-colored and, in places, waxy, glistening papular eruption was present over the anterior and lateral thoracic regions and upon the extensor surfaces of the lower extremities.

A case analogous to this one, Prof. Kaposi stated he had never seen, nor is there any similar instance recorded in the literature of lichen rubor planus, complicated by pemphigus vulgaris.

This patient continued in a precarious condition for some time, finally showed gradual signs of improvement, and was alive when I left Vienna.

The treatment of the case was symptomatic, and consisted, for the most part, in the use of stimulants and restoratives. The arsenic, which had previously been given for the lichen, was discontinued.

I observed a number of other interesting cases of pemphigus. At one time eleven patients suffering from this disease were presented within fourteen days. It is proper to state that a few of these were walking cases, living in the vicinity, which were sent for by the professor while he was treating the subject in his lectures. At all times, however, there are from one to one-half dozen serious cases of pemphigus lying in bed in the wards, and the suffering which these patients must endure with the more or less extensive denudation of the cutaneous surface by the large blebs which form, is very intense. Perhaps, in addition to this, there are extensive pemphigus patches upon some part of the mucous surface, especially the mouth, so that even the natural processes of mastication and deglutition distress the patient, and his condition is altogether miserable. His immediate comfort is not much added to by the water bath, in which he must lie for two to five hours each day, in accordance with the rule of treatment here enforced. I refer to the so-called "permanent bath" which originated with Hebra in the treatment of severe cases of variola. All phases and varieties of pemphigus have been observed. One case of particular interest, to which I wish to refer, is that of a woman, 67 years of age, who was suffering from a dark, pigmentary discoloration of the skin in the axillary regions and over the inner surface of the thighs. There was very deep induration and some pain. The duration of the disease was nine months. The patient's history was negative. This was a case of "pemphigus papillaris vegetans" (Neumann), which is usually characterized by the rapid development of a fungoid growth that in places becomes necrotic and gives rise to a rich viscid secretion. This condition may continue for some time or a rapid serpiginous extension take place, with the formation of vesicles, and in this way the development of a large raw surface. Pemphigus which assumes the variety here outlined is usually fatal in the course of a few months, the patient failing rapidly from loss of the serum of the blood, or he may die as the result of some complication, as nephritis, or edema of the lungs, etc. In the case here cited, the course seemed unusually slow and none of the above grave symptoms presented themselves. Where reformation of the epidermis takes place with dark pigmentation, such as was noted in this instance, recovery is not uncommon. The patient was doing well at the time I last saw her.

Such pemphigus papillaris vegetans areas occurring about the external genitalia, which is their common site, may be mistaken for certain venereal affections, such as condylomata, for a time, but with the appearance of similar pemphigus areas over other parts of the body the diagnosis becomes easy. Ample clinical material for the study of the various other phases of pemphigus, the acutus, hemorrhagic, serpiginous, pemphigus vulgaris, etc., was presented.

Rhinoscleroma is a disease which, while not exclusively confined to Austria-Hungary, is much more common in that country than in any other part of the world. A considerable number of cases, however, is observed in Italy. Its name implies, in a considerable measure, its character and location. The disease usually begins about the alæ of the nose, on the mucous surface, and in a very insidious manner extends backward through the posterior nares to involve the pharynx and upper respiratory apparatus. It may remain confined to one of these localities. There is great vascularity, and cellular infiltration of the tissues of a somewhat sarcomatous nature histologically, but with no such tendency clinically. Extreme induration takes place, as the suffix *sclera* implies. This is owing to the increased development of connective tissue. The part becomes dry, pale and hard, and, of necessity, the function, the secretion, etc., of the mucous membrane is seriously interfered with and finally completely abolished. The nasal orifices become encroached upon by the growth, and breathing through the nostrils is impeded. These patients usually snore intensely. With extension of the disease to involve the larynx, the voice becomes modified and assumes a nasal twang. The Eustachian tube is sometimes invaded, and the disease may find its way through to the external auditory meatus. Sometimes large erosions form and perforation of the part affected takes place. The course of this disease is exceedingly *chronic.* It lasts, in individual instances, for ten to twenty years. Three cases were under treatment continuously during my stay in Kaposi's clinic, and I saw several additional cases in Prague. One of the former was that of a woman, who had had the disease for a long time, and it had extended from the nostrils to involve the posterior nares and the upper part of the larynx. Her voice had changed considerably. She had become quite hoarse. The tissues presented a very pale, sclerotic appearance, much resembling scar tissue. The membrane of the nostrils had become as firm as cartilage. The diagnosis of this disease can usually be made on sight. Very little is accomplished by treatment, as a rule. Pyrogallic and 1 per cent. sublimate lanolin ointment have, in some instances, proved effective.

The cause of rhinoscleroma is now quite firmly established. A bacillus was first discovered in the newly-formed tubercles of this disease, by Fritch, in 1882. His observations have since been corroborated by a number of bacteriologists. Paltauf was able to demonstrate the presence of the germ in a series of seventeen cases studied, and, in 1886, he, as well as von Viselsberg, obtained *pure* cultures of it. This organism is a short thick bacillus with rounded ends, and is enveloped in a gelatinous membrane much resembling the capsule of Friedlander's bacillus. It is erobic, non-motile, non-liquifying, stains readily with the aniline dyes and may also be stained in the tissues. Its growth upon agar and gelatin much resembles the Friedlander bacillus, with which it is thought by many to be closely related. Inoculated into mice and guinea pigs, the bacillus proves pathogenic, although the typical pictures of rhinoscleroma have not thus been produced.

A very exceptional case of ichthyosis hystrix was seen in a child, four years of age, in which the body and extremities were completely covered with veritable spines of horny growth. Most of such cases, I believe, are congenital, and we have the one instance in which a whole family, composed of several members, were affected with this disease. They traveled over all Europe, at the beginning of the present century, as objects of curiosity, and were known as the porcupine men. The name of this family was Lambert. The individuals actually resembled porcupines, as did the child to which I here refer. The epidermal accumulations upon these patients sometimes reach the height of a centimeter, and may be still larger. Extensive desquamation is usually in progress, and the bed in which the patient lies is found covered with scales and broken-off masses of horny substance. Usually, the skin becomes intensely pigmented, so that the appearance of the individual is very striking and characteristic.

I desire next to record a most peculiar and unusual case of psoriasis, which I had the opportunity of seeing in Pick's clinic, and the character of which I do not think is duplicated. The anomaly, so to speak, of this case consisted in the formation of tumors, varying from the size of an almond to that of a walnut, scattered irregularly over the surface, and corresponding with what in ordinary cases are the flat patches of psoriasis. The pathology of the process must have been essentially the same as that of ordinary psoriasis. The usual overgrowth of the inter-papillary zones of the rete and the corresponding increase in the length of the papillary portion of the corium which separates them, was simply carried to an extreme degree, resulting in the formation of the large growths which I have described.

Further proof of the case being psoriasis in nature was the therapeutic. The firm papillary enlargements upon the surface subsided promptly under arsenic. Continuance of this remedy in full doses finally led to complete recovery.

With the vast amount of material which is present in Kaposi's wards, the various changes in the clinical course and morbid anatomy of psoriasis may be studied amply. The diffuse and universal eruptions and the punctate, psoriasis figurata, guttata et gyrata, psoriasis annularis, etc., are here seen beautifully illustrated. In no instance, however, have I observed anything that even simulated this case which presented the large tumor formations.

The preferred treatment in psoriasis, as regards the local application, at the time when I was with Kaposi, consisted in the use of gallacetophenone, a derivative of pryogallol, which appears in form of a yellowish crystalline powder, that is soluble in hot water, alcohol, ether and glycerine. This remedy is applied in 10 per cent. solution and repeated every few days. It is penciled over the affected areas and confined to them. The patient's body, after such a treatment, appears very picturesque ; he is spotted somewhat like a leopard. The yellowish areas of varying size stand out in striking contrast to the remainder of the unaffected cutaneous surface.

The number of cases of lupus which were under treatment daily in the clinic over a period of several months, averaged about thirty. By no means all of these were confined to the face. In several instances the hands and other parts of the body were the seat of disease. Three cases of complete destruction of the nose, the result of recurrent lupus vulgaris, were in the hospital. A fresh case, coming under Prof. Kaposi's observation, is immediately scraped to the very depths of the infiltration with a sharp spoon curette. Cocaine is injected sometimes, but, as a rule, the time for this luxury to the patient is not taken, and then the individual has to suffer. After all of the diseased tissue is removed, pyragallol salve, or some other cauterizing agent, is applied, and the part dressed with simple ointment until recovery. As a rule, after this radical treatment, more or less scarring takes place. When a new area happens to start up it is subjected to the same course of treatment, being first thoroughly *scraped.*

In contrast to this procedure for lupus, interesting results from the use of tuberculin, under Prof. Pick, in Prague, may be seen. This is his universal treatment, and uniformly recovery takes place. The injection of about 1-130 of a grain is followed in twenty-four hours by the characteristic reaction, and, in this instance, it can be plainly seen to occur at the location of the disease. A breaking down and gradual extrusion of the tubercular tissue takes place, and then the part heals by granulation. The tubercle bacilli are more readily found in the affected tissue after an injection of the lymph, the tubercular process having been lighted up. At other times, as one who has tried it can attest, the preparation of a vast number of sections is often necessary to demonstrate the presence of the tubercle bacillus in lupus tissue. Again, it may be a comparatively easy task.

As somewhat allied in the character of their etiology to the disease now under consideration, I might refer to two cases of leprosy, which were under treatment in the wards of one of the clinics in Vienna at the time that I was there. These cases were represented in two girls, who came from the Island of Java and were sisters. Their ages were seventeen and twenty respectively. They had been affected with the disease for some time, just how long I cannot state. Their cases were of the *anesthetic* type. The principal objective symptom was marked and extensive pigmentation (dark) of the affected areas of the body and extremities. There were no necroses and ulcerations with dropping off of phalangeal members, like we read about, and, to illustrate the non-transmissibility of leprosy under most circumstances, these two cases were allowed to live in the ward and associate with the twenty or thirty other patients. Few, if any, of the latter knew that those two had the disease from which they were suffering. None of the other patients acquired it during the six months which followed the arrival of the lepers. The latter were treated with arsenic, continued to improve, and were doing well when I left Vienna.

The disease prurigo, so common in Vienna, and which I considered of particular interest, owing to the discussion which exists in regard to it, a case of frœmbesia syphilitica, several cases of lupus erythematoides, and a certain other case of interest, I hope to treat further, under the head of this paper, at some future time.

469 Delaware Avenue.

